# Mechanistic role of transglutaminase-2 in focal adhesions

**DOI:** 10.1038/s41598-018-30172-8

**Published:** 2018-08-17

**Authors:** Evelyn Png, Aihua Hou, Louis Tong

**Affiliations:** 1 Ocular Surface Research Group, Singapore Eye Research Institute, Singapore, 168751 Singapore; 2 Department of Cornea and External Eye Disease, Singapore National Eye Center, 11 Third Hospital Avenue, Singapore, 168751 Singapore; 3 Duke-NUS Graduate Medical School, Singapore, Singapore; 4 Yong Loo Lin School of Medicine, National University of Singapore, Singapore, Singapore

## Abstract

Transglutaminase (TG)-2 interacts with matrix proteins and integrins, forming focal adhesions (FA) to initiate cell migration, thus playing a vital role in wound healing. Previously we showed that TG-2 influenced phosphorylation of paxillin and other FA proteins. Here, we aimed to investigate the molecular mechanism of TG-2 regulation of paxillin. Human corneal epithelial cells expressing shRNA against TG-2 (shTG) and scrambled sequence control (shRNA) were cultured. TG-2 was pulled down by anti-paxillin antibody, but not MAP3K12. Cell-free interaction assay with immobilized paxillin shows that TG-2 bind to paxillin directly. JNK was the strongest kinase for paxillin phosphorylation in the *in-vitro* kinase screen, but TG-2 could not phosphorylate paxillin directly. Increasing TG-2 concentrations did not increase the amount of JNK in the TG-2/paxillin complex. Immunofluoresent staining shows that TG-2 colocalises with vinculin and paxillin in FA of migrating cells. TG-2 binds to paxillin and JNK-containing FA but does not recruit JNK directly. Taken together with previous findings, TG-2 binds paxillin non-covalently, and JNK can phosphorylate paxillin, these processes critically regulate corneal epithelial adhesion and migration.

## Introduction

Transglutaminase (TG)-2 is a ubiquitous multi-functional protein that plays a vital role in wound healing, cancer and development^1–3^. TG-2 can interact with ECM proteins and cell surface integrins during cell adhesion and migration processes^4–7^; thus forming structures called focal adhesions (FA)^8,9^ linked to cellular cytoskeleton that can initiate and propagate cell movement. FA are multi-protein structures^10^ that forms and matures progressively upon the sequential recruitment of adaptor and signalling proteins such as integrin, talin, paxillin, vinculin and focal adhesion kinase (FAK)^11^.

However, the factors affecting the robustness of recruitment and association between various adhesion proteins are not well studied. TG-2 has been reported to activate integrins, Rho or FAK^6,9,12^, thus we speculate that it may also promote recruitment or binding of adhesion proteins within the FA. One such adhesion protein is paxillin, a 68 kDa adaptor protein important for coordinated recruitment and activation of other proteins^13^. Paxillin is indispensable for life, as deletion of paxillin gene leads to defective cell migration and cell spreading during development^13,14^ and hence embryonic lethality.

Previously we demonstrated that TG-2 status of corneal epithelial cells is linked to the phosphorylation of serine 178 in paxillin^15^. In addition, the phosphorylation at this position has been shown to be necessary for normal adhesion and migration^16^. However, it is not known how TG-2 influences the phosphorylation of paxillin. Potentially this can be a direct (covalent, non-covalent, enzymatic) or indirect mechanism^17^. The mechanism may or may not involve recruitment of additional kinases to the FA^17^.

We hypothesize that TG-2 directly binds to paxillin. Here, we show that TG-2 interacted non-covalently with paxillin, and propose a model where interaction of TG-2 with paxillin facilitates phosphorylation of paxillin and other adhesion proteins and maturation of the adhesion complex.

## Results

### TG-2 binds to paxillin by non-covalent interaction

**Figure 1:**
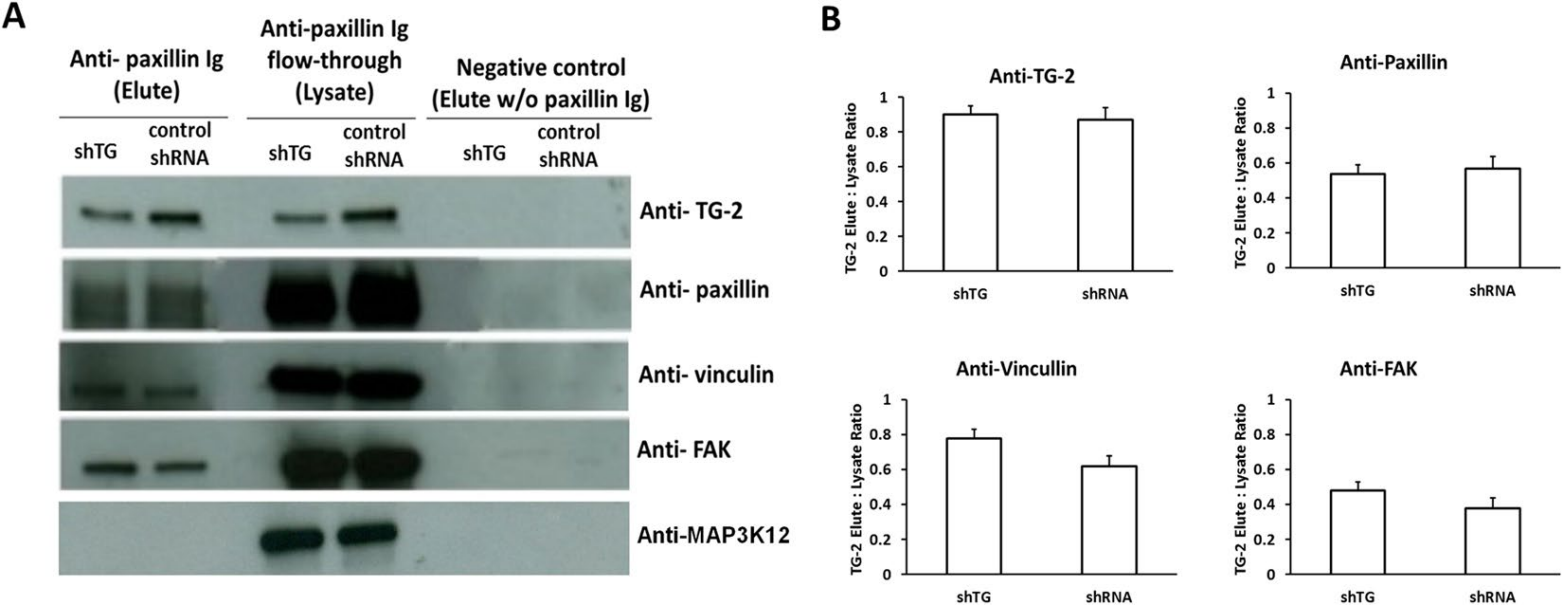
Co-immunoprecipitation of shTG and control cell lysate against anti-paxillin. (**A**) Total cell lyate from cell line shTG and control shRNA was immunoprecipitated with or whithout anti-paxillin. Elute and flow-through was used for western blotting with antibodies against TG-2 and focal adhesion proteins: paxillin, vinculin and FAK. Elute from plain streptavidin agarose beads without adhering antibody was used as negative control. (**B**) Bar chart showing the densitometric quantification of the proportion of proteins pulled down with paxilllin, using paxillin antibody, from the lysate in shTG and shRNA cell line. Height of bars represent mean of ratios of 3 experiments, error bars indicate standard deviation. U tests were used to compare the differences between heights of the 2 bars.

Co-immunoprecipitation data show that TG-2 was successfully pulled down using anti-paxillin antibody in both shTG and shRNA cell lysates (Fig. 1). Control cells contained more TG-2 in these complexes, compared to shTG (Fig. 1 columns 1 and 2, row 1), even though both cell types express the same amount of paxillin (Fig. 1 row 2 columns 3, 4) and similar amounts of paxillin were pulled down in the assay (Fig. 1 row 2 columns 1, 2). Apart from immunobloting, the gel fragment containing the band at approximately 70 kDa was excised and mass spectrometry showed that this band contained human TG-2 sequences (data not shown).

Vinculin and FAK were also co-immunoprecipitated by anti-paxillin antibody (Fig. 1 rows 3, 4), suggesting that these are *bona fide* mature focal adhesion complexes, whose components separated under denaturing conditions. The amount of TG-2 did not affect the binding of FAK or vinculin to the paxillin-containing complex (Fig. 1 comparing columns 1 and 2 rows 3, 4).

As TG-2 is a very ubiquitous cellular protein present in many subcellular compartments^18^, it is necessary to show that the binding does not occur indiscriminately. We confirmed that TG-2 did not bind to an irrelevant protein (MAP3K12) which is present in the lysate flow through (negative control, last row Fig. 1). Furthermore there was absence of any higher molecular weight bands to suggest covalent binding of TG-2 to paxillin (data not shown).

**Figure 2:**
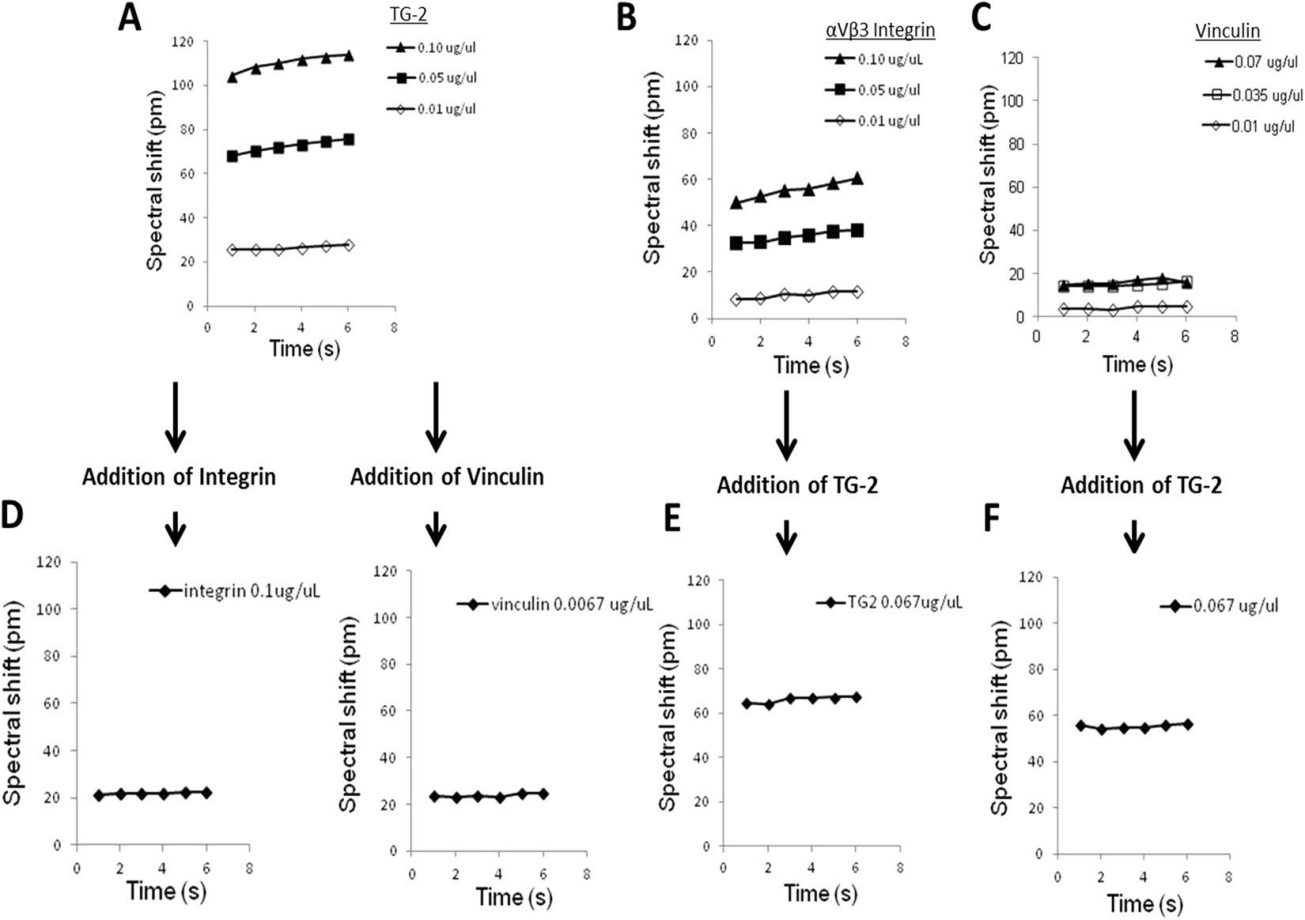
Label-free biochemical assay with optic grating sensor technique. Recombinant human paxillin was first immobilized at a final concentration of 0.05 µg/µL on the amine-coupling surface of microplate. After the immobilized paxillin were in equilibrium, the spectral shift was reset arbitrarily to zero picometers (pm) before adding a second recombinant protein, either TG-2, αVβ3 Integrin or Vinculin, at the indicated concentrations. The spectral shift was then measured and recorded by Enspire Multimode Plate Reader (Top row: **A**–**C**). These shifts were again reset to zero in the wells that no significant interaction of the added protein with immobilised paxillin, and 15 µl of the third recombinant protein were added to those wells and the spectral shift was then measured and recorded (Bottom row: **D**–**F**). Experiment has been repeated twice.

TG-2’s interaction with paxillin was confirmed in an *in vitro* cell-free system (Fig. 2A). When different concentrations of TG-2 and αVβ3 integrin recombinant proteins were added to the wells pre-coated with paxillin protein, significant spectral shifts were detected after equilibrium, suggesting TG-2 (Fig. 2A) or αVβ3 integrin (Fig. 2B) can individually bind to the paxillin. In contrast, vinculin binding to paxillin was not conclusive (Fig. 2C) as there was minimal spectral shift.

In the context where TG-2 already interacted with paxillin, addition of vinculin (Fig. 2D right) or αVβ3 integrin (Fig. 2D left) caused additional interactions, which may be due to these two molecules binding to TG-2/paxillin complex, or that the two additional molecules enhancing the pre-existing TG-2/ paxillin interaction.

After addition of TG-2 to the integrin/ paxillin containing solution (Fig. 2E), there was further increase in spectral shift. This implied that either the newly added TG-2 binds to the integrin/ paxillin complex, or that it increased the binding of previously added integrin to immobilised paxillin.

When TG-2 was added subsequent to the addition of vinculin, a significant spectral shift occurred. This suggested that newly added TG-2 has bound to the immobilised paxillin (Fig. 2F), suggesting that TG-2 paxillin interactions are stronger than any vinculin paxillin interactions. Experiments repeated at pH 4.3 showed similar results (data not shown).

**Figure 3:**
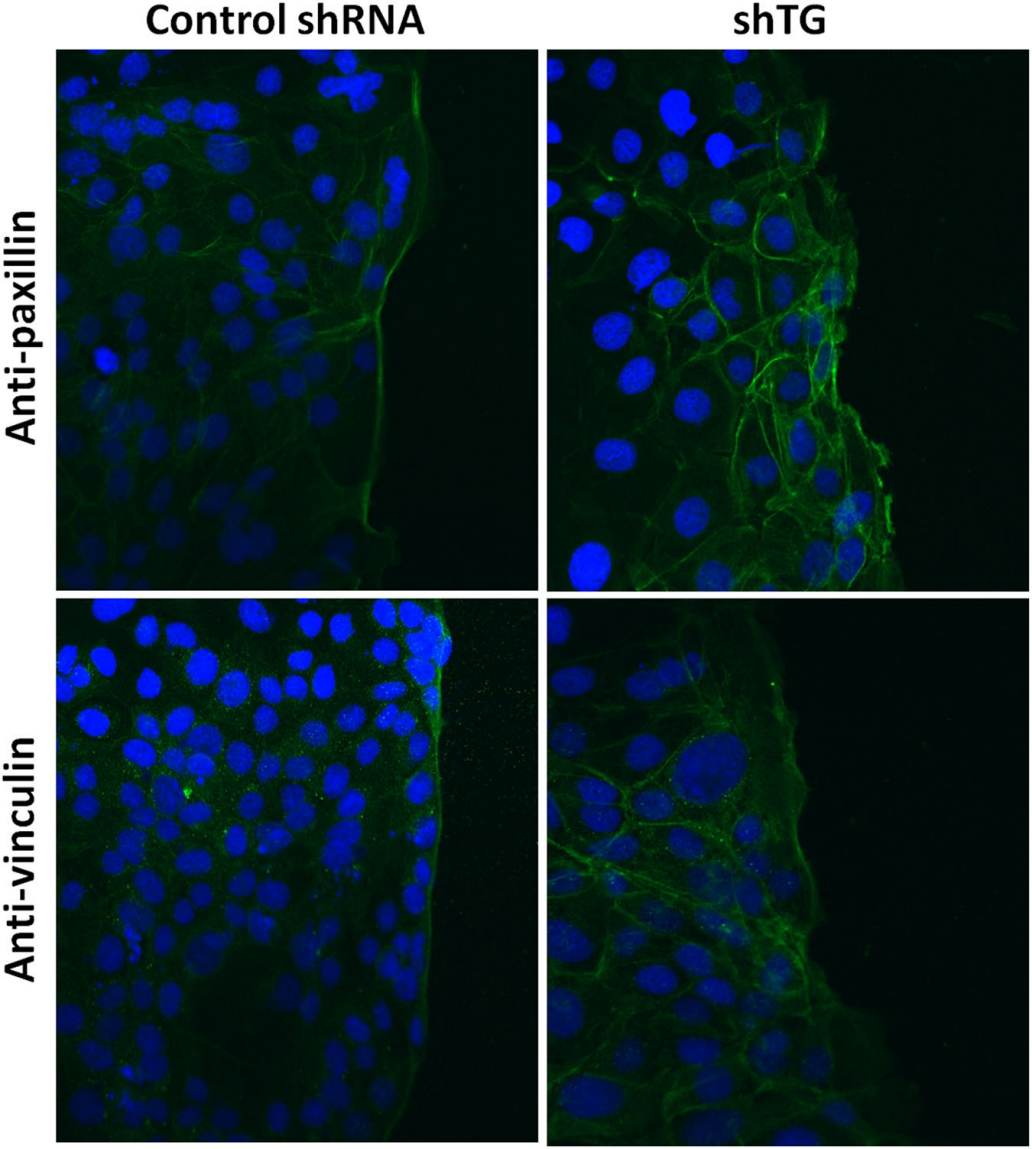
Immunofluorescent staining of paxillin and vinculin in control shRNA and shTG cells. Cells were seeded into chamber slides and cultured until confluent. A scratch was made through the cell monolayer and cells were cultured in fresh medium for 2 hrs before immunofluorescence staining was performed. Green: Paxillin and vinculin; blue: DAPI stained nuclei. Total of 3 replicates have been performed. Scale bar = 50 μm.

In order to examine if the above interactions occur within intact cells, immunofluorescent imaging of vinculin and paxillin proteins was performed (Fig. 3). In control cells, vinculin and paxillin were localised in a linear advancing cell edge as a relatively straight and well-defined line whereas in shTG cells, the advancing complexes were disrupted, so this line was not visualised. These findings suggest that TG-2 interact with vinculin and paxillin in focal adhesions located in intact cells.

### JNK but not TG2 phosphorylates paxillin in cell free system

Because of our previous findings that TG-2 status was associated with phosphorylation of paxillin at Ser178, (CITE BBA) we screened a kinase library that may phosphorylate paxillin directly. The highest absolute kinase activities in the assay were (in decreasing activities) microtubule affinity regulating kinase (MARK)3, glycogen synthase kinase (GSK)3-alpha and MARK1 protein kinase. However, the highest relative activities in decreasing order (which removed the autophosphorylation effects of the kinases on themselves) were obtained for c-Jun N terminal kinase (JNK)1, protein kinase (PK)A and MARK2 (Table 1).

**Table 1:** Serine/Threonine Kinases.

Kinase	A	B	Kinase autophos, SD	C	Activity values, corrected (A–C)	Activity Ratio (A–C)/B
	Activity raw values	Kinase autophos, normalized mean n = 3		Substrate-BG, mean of 2 singlicates		
JNK1	3986	**43**	33	394	**3592**	**84.34**
PKA	7600	**87**	14	394	**7206**	**82.43**
MARK2	24606	**330**	34	394	**24212**	**73.30**
DYRK1B	6317	**81**	63	394	**5923**	**73.12**
NEK7	22181	**304**	86	394	**21787**	**71.64**
PRKX	5872	**77**	51	394	**5478**	**71.14**
GRK2	16225	**232**	83	394	**15831**	**68.17**
MAPKAPK3	1632	**18**	2	394	**1238**	**67.28**
TSSK1	14094	**204**	134	394	**13700**	**67.25**
GRK3	6065	**112**	2	394	**5671**	**50.75**
CDK5/P25NCK	13798	**265**	66	394	**13404**	**50.62**
CK1-gamma2	14823	**300**	18	394	**14429**	**48.15**
CDK2/CycA	27128	**621**	40	394	**26734**	**43.03**
CK1-delta	12435	**281**	24	394	**12041**	**42.86**
IKK-epsilon	26037	**619**	42	394	**25643**	**41.41**
TBK1	18074	**431**	37	394	**17680**	**41.03**
TSF1	11932	**282**	93	394	**11538**	**40.89**
CK1-epsilon	9899	**250**	59	394	**9505**	**38.03**
CDK7CycH	11528	**296**	97	394	**11134**	**37.60**
MINK1	6843	**183**	90	394	**6449**	**35.22**

**Figure 4:**
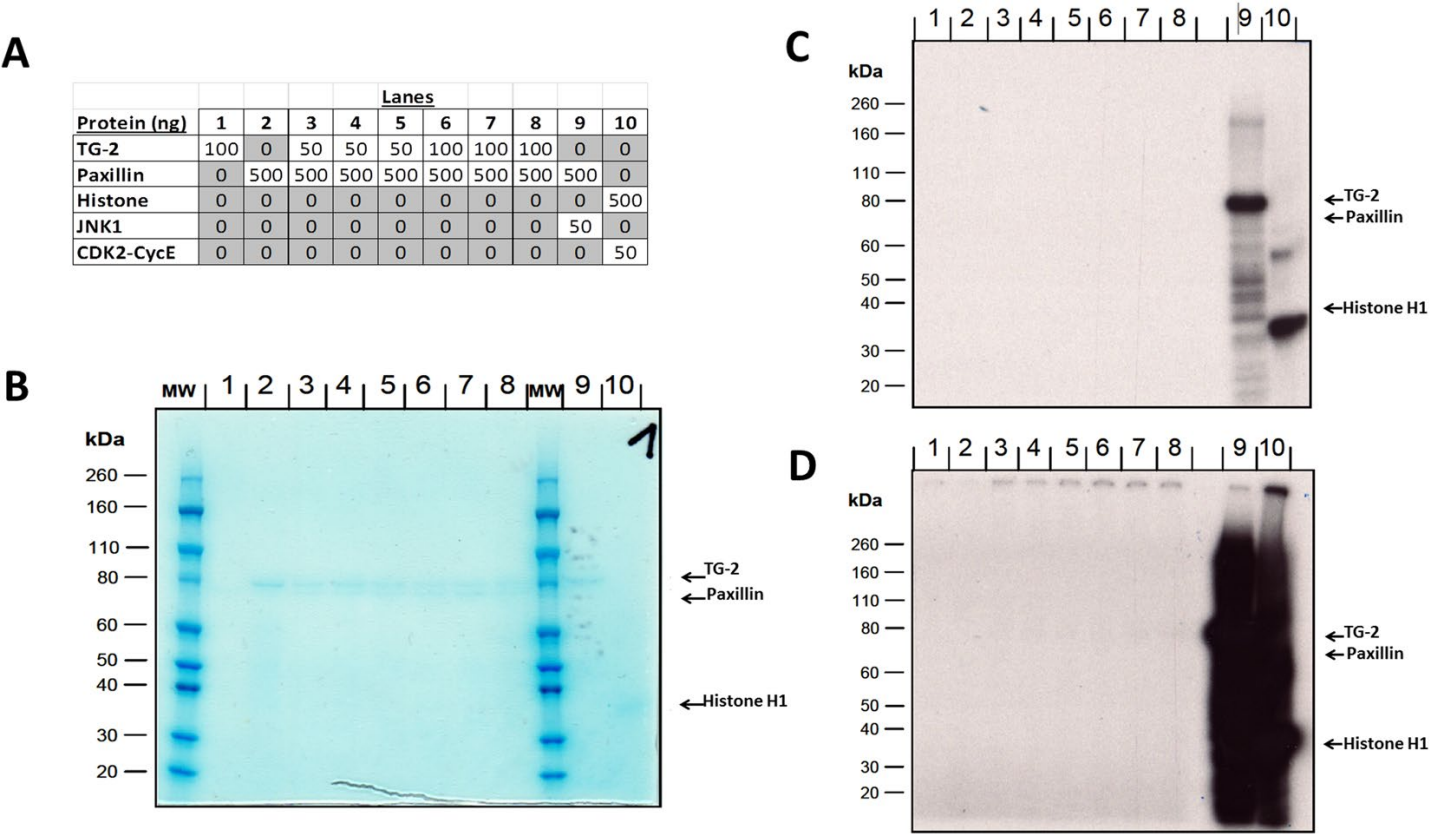
*In-vitro* kinase assays. (**A**) Paxillin and different concentrations of TG-2 were loaded into lane 1–8 of a 4–12% SDS-PAA gel. JNK is paxillin positive kinase control (lane 9), histone and CDK2-Cyce is the positive control of the kinase assay. (**B**) Coomassie blue staining after gel running. (**C**) Autoradiogram image after the gel was dried and exposed to x-ray film for 60 min. (**D**) Autoradiogram image after the gel was dried and exposed to x-ray film for 3 days. MW = molecular weight marker. Experiments have been repeated three times.

To evaluate whether TG-2 itself can phosphorylate paxillin, an *in vitro* kinase assay was carried out (Fig. 4). The amounts of paxillin, TG-2 and the control kinases loaded on the SDS-PAGE gel (Fig. 4A) were just detectable by coomassie blue staining on the gels (Fig. 4B). In the autoradiogram (Fig. 4C, lower arrow), a strong band could be detected for the positive control at the expected molecular weight of Histone H1 in lane 10 (CDK2-CycE1 phosphorylation of Histone H1), demonstrating the previously reported ability of CDK2-CycE1 to phosphorylate Histone H1^19^.

To confirm that JNK could phosphorylate paxillin as discovered in the kinase screening assay, we repeated this reaction in the experiment. We confirmed that JNK phosphorylated paxillin, illustrated by the band which contained radioactive paxillin in lane 9 (Fig. 4C, higher arrow).

Auto-radiographic signals could not be detected for paxillin or TG-2 in Fig. 4C lanes 1–8. TG-2 neither phosphorylates paxillin (Fig. 4C, lanes 3–8) nor auto-phosphorylates itself (Fig. 4C, lane 1) under these conditions. Also, no protein phosphorylation activity could be found for the paxillin preparation alone, showing that paxillin could not phosphorylate itself in these conditions (Fig. 4C, lane 2). Even after a significantly longer 3 day exposure of the radioactive gel, no appreciable signal for either paxillin or TG-2 could be detected (Fig. 4D). In summary, JNK but not TG-2, phosphorylated recombinant paxillin strongly in a cell free system.

### Recruitment of JNK is TG-2 independent

**Figure 5:**
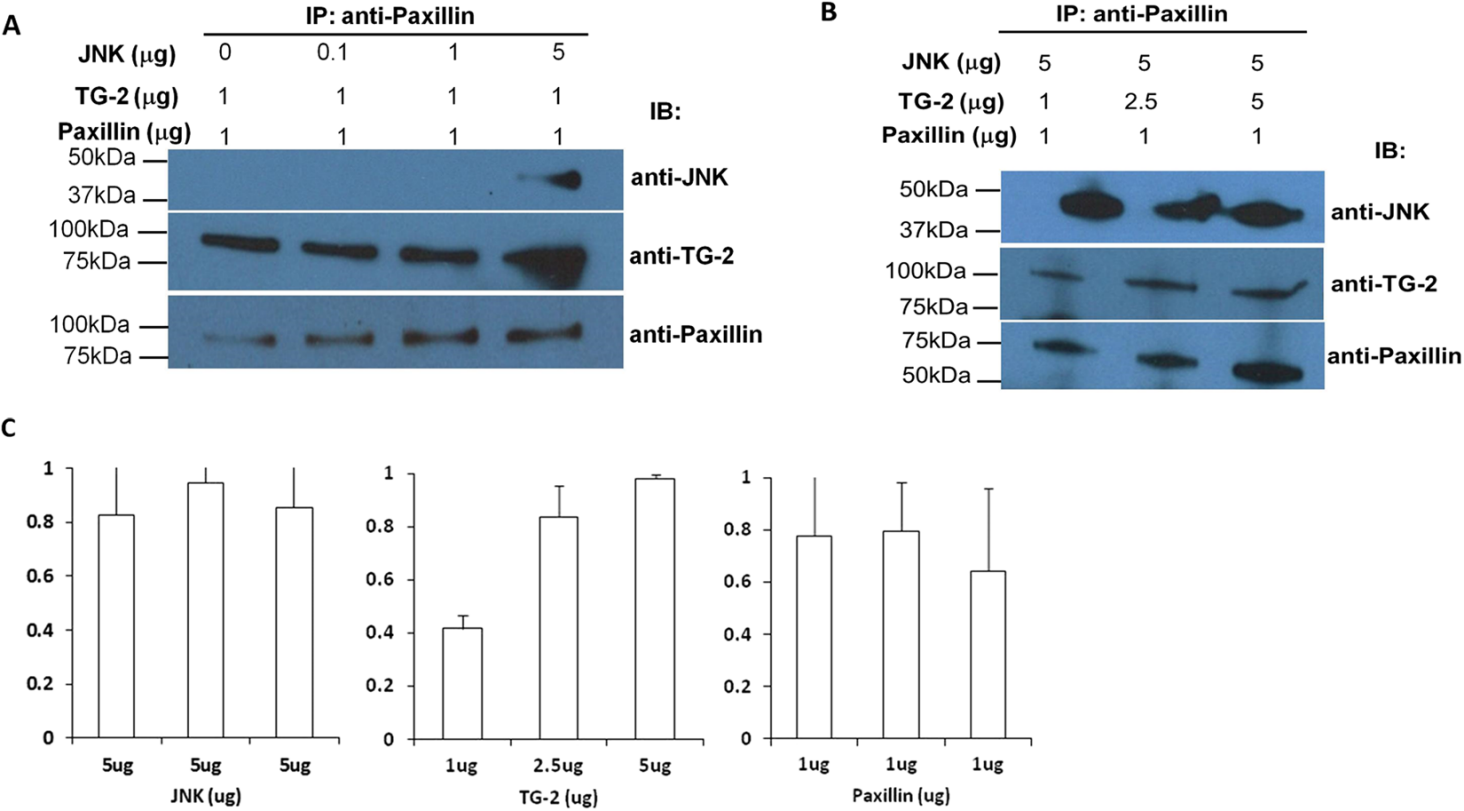
Cell free *in-vitro* immunoprecipitation and western blotting. Immunoprecipitation was performed using a combination of human recombinant proteins TG-2, JNK and paxillin. These recombinant protein mixtures were incubated at 4 °C overnight and immunoprecipitated with anti-paxillin. Western blot was then performed using the pulled down proteins with anti-TG-2, anti-JNK and anti-paxillin. (**A**) Increasing concentration of JNK protein at 0, 0.1, 1 and 5 µg were added with fixed amount (1 µg) of TG-2 and paxillin respectively. (**B**) The amount of JNK and paxillin were fixed with 5 µg and 1 µg respectively, while increasing concentration of TG-2 at 1 µg, 2.5 µg and 5 µg was used per reaction. (**C**) Bar chart showing the densitometric quantification of the proportion of JNK, TG-2 and paxillin proteins pulled down using paxillin antibody. Height of bars represent mean of ratios of 3 experiments, error bars indicate standard deviation. One way ANOVA was used to compare the differences between heights of the 3 bars.

Previously, it was found in corneal epithelial cells that S178 phosphorylation of paxillin was JNK-mediated^16^. We were interested to determine if TG-2 could affect recruitment of JNK to the complex. First we performed immunoblots against recombinant TG-2, paxillin and JNK at various concentrations to ensure that these could be detected. We then confirmed with co-IP that recombinant TG-2 can bind to paxillin complexes, which also bound JNK (Fig. 5). This IP was evaluated using 3 concentrations of JNK (Fig. 5). When TG-2 concentration was increased in the presence of fixed concentrations of paxillin and JNK (evaluated in the preceding experiment), there was no corresponding increase in the intensity of JNK in the immunoblots, suggesting that the amount of JNK in the complex did not increase in a TG-2 dependent manner (Fig. 5).

## Discussion

In this study, we found that TG-2 can bind to paxillin in cell lysates and pure protein solutions (immobilised or free in solution), and within intact cultured cells. This complex is likely a cell adhesion complex because it also contains classical adhesion proteins like vinculin and FAK. The interaction between TG-2 and paxillin is likely non-transamidase dependent, as it occurred even between recombinant proteins *in vitro*, where the conditions were not favourable for enzymatic catalysis. We also showed that even though JNK can phosphorylate paxillin, it was not recruited to the complex in a TG-2 dependent way.

We previously found that TG-2 is necessary for phosphorylation of paxillin at S178^15^, and currently we confirmed that JNK is a strong kinase for this reaction and that TG-2 cannot phosphorylate paxillin by itself. This implied that TG-2 has facilitated the kinase activity of JNK, or increased the propensity of paxillin to serve as a substrate for phosphorylation. Previously we have also reported that TG-2 status affects phosphorylation of integrin beta-3, vinculin and FAK^15^. Therefore it is likely that once paxillin has been phosphorylated, the complex may be enabled to phosphorylate other members of the adhesion complex.

The cascade of phosphorylation events in the adhesion complex is necessary before a mature focal adhesion can anchor the cytoskeleton of the cell to matrix^11,20^. Since TG-2 is ubiquitously present in the cell cytosol, its non-covalent interaction with paxillin may help it to localize to the focal adhesion complex, and this can be a critical step that regulates the formation or maturation of the complex. It would be useful to evaluate if the TG-2 paxillin complex can increase the recruitment of other focal adhesion proteins.

We found through the *in vitro* screening assay that JNK is a very strong kinase for phosphorylation of paxillin. Therefore it is likely that JNK phosphorylates the paxillin in the adhesion complex. Consistent with our findings, another group has also found JNK to be a kinase present in the complex and that JNK can phosphorylate the paxillin. In cultured cells with serum free media, addition of the JNK inhibitor SP600125 (10 µM) for 24 hours resulted in reduction of S178 phosphorylation of paxillin, but not the expression level of paxillin^16^.

If JNK is the mediator that phosphorylated paxillin, what is the relationship between TG-2 and JNK? We showed that increasing TG-2 did not increase the JNK in the complex, which implied that TG-2 was not involved in recruiting JNK to the complex. Although it is known that TG-2 can crosslink and activate MAP3K12 and subsequently activate JNK^21,22^, we don’t think this specific mechanism is active in our cell context since the MAP3K12 is not part of the adhesion complex. Although we previously showed that TG-2 is necessary for the paxillin phosphorylation, we have not shown that the interaction with paxillin is necessary for JNK activation.

If TG-2 can bind directly to paxillin, a question would be the location of the binding sites on paxillin? We were unable to localise the binding with paxillin to one domain of TG-2^18^, but perhaps structural biology studies can help to elucidate the nature of the interaction better. We suggest that TG-2 may have interacted with the LD domains in the amino terminal region of paxillin to affect the recruitment of other proteins, since LD domains are known to have various protein binding sites^17^.

The biological significance of the current findings is that we have reported TG-2’s role to be indispensable in epithelial migration and wound closure in cornea wound healing^15^, and another group has confirmed that the S178 phosphorylation in paxillin is vital for corneal epithelial cells to execute such functions^16^. Since wound healing is commonly disturbed in ocular surface diseases and inflammation, the interface between TG2 and paxillin may be a potential therapeutic target. Increasing the strength of this interaction may facilitate cell adhesion and wound closures, whereas disrupting these interactions may cause detachment and loss of survival of cells in disease conditions. These speculations can be explored in future studies.

The main strength of this paper is the confirmation of the interaction between TG2 and paxillin. Although we do not have direct evidence that this interaction is important for JNK to be activated, when our data and previous experimental data are considered collectively^15,16^, the localisation of JNK to the complex, the affinity of JNK for paxillin and the effect of inhibiting JNK on paxillin phosphorylation suggest that JNK may indeed be the kinase that TG2 induces for its effects in this context. The downstream action of JNK on the focal adhesion complex may involve other players, is not a focus of this paper and should be evaluated further.

In conclusion, we showed that TG-2 is a novel adhesome protein that interacts with paxillin directly and the binding also occurs in whole corneal epithelial cells. Taken together with results of related research, this is an important and novel finding concerning the basic mechanism of epithelial cell adhesion and movement, with implications for diseases involving defective wound healing.

## Materials and Methods

### Cell lines

Stably transfected cell lines shTG or shRNA were described previously^18,23^. In summary, shTG cell line were generated by stably transducing human SV-40 immortalized corneal epithelial cell line HCE-T (RCB 1384, Riken Cell Bank, Ibaraki, Japan) with shRNA targeting TG-2; and control HCE-T cells were stably transduced with non-specific scrambled shRNA (referred to as ‘shRNA’ in short). Cell lines were maintained in DMEM-F12 supplemented with 5% FBS at 37 °C in a humidified incubator with a 5% CO_2_. Medium was replaced every the other day. These cell lines were recently authenticated and they were not contaminated.

### Chemicals and reagents

PBS was from Nacalai Tesque (Kyoto, Japan). RIPA buffer, Bovine serium albumin (BSA), protein inhibitors and phosphatase inhibitors were purchased from Sigma-Aldrich (St. Louis, USA). Streptavidin agarose was from Pierce (IL, USA). Fetal bovine serum (FBS) was from Life Technologies (California, USA). Recombinant paxillin for kinase assays and cell free immunoprecipitations were synthesized by Biolegend (California, USA). SDS-PAGE ready gel was obtained from Bio-Rad Laboratories (California, USA). Running buffer was a Tris/glycine/SDS running buffer from Biorad, (CA, USA). Transfer buffer was Tris/glycine transfer buffer from the same company with addition of 20% methanol, kept chilled before use. TBST wash buffer is composed of 0.1% Tween 20 in Tris buffered saline. Streptavidin agarose slurry was from Thermo Scientific Pierce® (IL, USA).

### Immunoprecipitation using shTG and shRNA cell lysates

The shTG and shRNA cells were cultured in serum free media on fibronectin-coated plates for 24 hrs. After washing the cells with chilled PBS, cell lysis was performed in RIPA buffer containing phosphatase and protease inhibitors. After spinning down, protein concentration was determined as previously described^18^. Briefly, 500 micrograms of total proteins was used in each condition or control. The lysate was first pre-cleared by incubating with 60 μL of streptavidin agarose slurry for 1 hr by end-over-end mixing, then the lysate and agarose mixture was centrifuged at 1000 g for 1 min at 4 °C. The supernatant was incubated with biotin-conjugated rabbit anti-paxillin (1:20, Bioss, MA, USA) and rotated continuously overnight at 4 °C. Next day, 60 µl of streptavidin agarose slurry was added to the mixture and the mixture was incubated at 4 °C for 2 hrs with end-over-end mixing, followed by centrifugation at 1000 g for 1 min at 4 °C. The beads were washed 4 times with binding buffer solution and boiled at 95 °C for 9 min with 50 µl of 2X protein loading buffer containing 20% reducing agent.

### Western blot

Immunoblotting was performed as previously described^23^. Briefly, 40 μg of protein was mixed with 4x loading dye containing 20% beta mercapethanol. The samples were denatured at 99 °C for 10 minutes. For identification of protein bands, 10 µL of protein standard with known molecular masses was loaded in one lane. The gel electrophoresis was carried out at 100 volts for approximately 1 hour and 30 minutes until the dye front reached the bottom of the gel. PVDF membrane was activated with 100% methanol for 15 seconds, followed by soaking in water for 2 minutes.

The elute proteins separated under denaturing conditions on the PAGE-gel electrophoresis were transferred to the activated PVDF membranes overnight at 20 v. After the run, the PVDF membrane was washed 3 × 5 minutes in TBST, followed by blocking in 5% Bovine Serum Albumin (BSA) in TBST for 1 hour at room temperature. This is followed by 3 × 5 minutes washing in TBST at room temperature with shaking.

Primary antibodies against paxillin, TG-2, vinculin, focal adhesion kinase, MAP3K12 were added into 1% BSA in TBST respectively and incubated for 1 hour at room temperature with shaking. This is followed by 3 × 5 minutes washing in TBST at room temperature with shaking. Horse radish peroxidase conjugated secondary antibodies targeting the species of the primary antibodies 1% BSA in TBST were incubated with the membranes for 45 min. This is followed by 3 × 5 minutes washing in TBST at room temperature with shaking.

Detection of chemiluminescent protein bands was performed with FEMTO substrate (Pierce, IL, USA), with the help of molecular weight standards (Bio-Rad, CA, USA). Primary and secondary antibodies used in this study were listed in Table 2.

**Table 2:** Antibodies used in the study.

Antibody	Source	Species	Cat. No.	Dilution factor
Anti-TG-2	Abcam	Rabbit	Ab421	1:1000
Anti-Paxillin	BD Transduction Laboratories	Mouse	610620	1:1000 for WB 1:100 for IF
Anti-vinculin	Abcam	Mouse	Ab18058	1:400 for WB 1:50 for IF
Anti-JNK	Sigma-Aldrich	Mouse	SAB4200176	1:1000
Anti-FAK	BD Biosciences	mouse	610087	1:200
Anti-MAP3K12	LifeSpan BioSciences	mouse	LS-C158096	1:1000
Anti-mouse HPRT	Sigma-Aldrich	Goat	A4416	1:8000
Anti-rabbit HPRT	Sigma-Aldrich	Goat	A0545	1:8000
Anti-mouse IgG FITC	Sigma-Aldrich	Goat	F2012	1:400

WB: western blot; IF: Immunofluorescence staining.

### Mass spectrometry

Anti-paxillin immunoprecipitated proteins from shTG and shRNA were separated by PAGE gel, commassie staining, in gel digestion and mass spectrometry of elute proteins were performed as previously described^18^. Briefly various bands of proteins with molecular weights corresponding to TG-2 and paxillin were excised from the gel. After in-gel tryptic digestion, peptides were analysed by nano LC/LC and MS/MS to sequence the peptide fragments. MASCOT software (Matrix Science, UK) was used for protein database search and identification of peptides.

### *In-vitro* protein interactions assay

The optic grating sensor technique, Enspire Multimode Plate Reader (Perkin Elmer, MA, USA), was used to evaluate *in-vitro* protein interactions. The readout of this assay measured changes in the index of refraction upon a binding event, indicated by a shift of wavelength of the incident light. Briefly, recombinant human paxillin (Biolegend, CA, USA) at a concentration of 0.15 µg/µL was immobilized on the amine-coupling surface of microplate at pH7.5 and incubated overnight at 4 °C. Next day, the non-binding paxillin was washed off with 30uL of pH7.5 PBS for three times. After adding 15 uL of pH7.5 PBS, the plate was read and and the spectral shift was reset arbitrarily to zero. Fifteen microliters of recombinant human TG-2 (R&D, Minnesota, USA), human beta-3 integrin (R&D MN, USA) or vinculin (Abnova, CA, USA) at concentrations ranging from 0.0067 to 0.1 µg/µL was added to the respective wells of the microplate. This is then followed by incubation of the microplate in the Enspire Multimode Plate Reader at 27 °C for 2 hrs. The microplate was washed three times with PBS and then the spectral shift was measured and recorded. In the scenario where there was no significant interaction of the added protein with immobilised paxillin, 15 µL of a third recombinant protein was added into each well and the assay repeated. The above experiments were also repeated at pH 4.3.

### Immunofluorescence staining

ShRNA or shTG cells were seeded into chamber slides and grown in DMEM-F12 media supplemented with 5% FBS for 24 hrs to produce a confluent monolayer of cells. Straight scratches were made with a 1 ml blue pipette tip in the monolayer. Cells were washed twice with PBS and continue cultured with DMEM-F12 media with 5% FBS for 2 hrs with fresh medium. Immunofluorescence was then performed as described previously^15^. Briefly, cells in chamber slides were fixed with 4% paraformaldehyde (PFA) for 15 min, permeabilised with 0.1% of Triton X-100 for 10 min and blocked with 1.5% BSA for 1 hr in humidified chamber. The slides were then incubated with primary antibodies (Table 2) in 1.5% of BSA for 2 hrs at room temperature. After washing off primary antibodies, secondary antibodies (Table 2) in 1% BSA was added to the slides and incubated in dark at room temperature for 1 hr. After washing 3 times with PBS, The slides were then mounted with mounting medium which containing DAPI (Santa Cruz, Texas, USA) and images were taken by Zeiss microscope (Zeiss, Oberkochen, Germany).

### Paxillin kinase screening assay

Paxillin kinase screening was performed through the KinaseFinder service provided by ProQinase (Freiburg, Germany). Human recombinant paxillin was prepared in a HEPES buffer and was tested at 5 μg/50 μl final assay concentration in a radiometric filter assay on a panel of 190 known Ser/Thr kinases (protein symbols shown in Table 1 first column).

The phosphorylation reaction cocktails were pipetted into V-shaped 96-well microplates in the following order: 10 μl of kinase solution, 40 μl of buffer/^33^P-γ-ATP/test sample mixture. One well of each plate was used for a buffer control without enzyme. The plates were incubated at 30 °C for 60 min before the reactions were stopped by 20 μl of 10% (v/v) H_3_PO_4_. Subsequently, the reaction cocktails were transferred into 96-well glass-fiber filter plates which were pre-wetted with 150 mM H_3_PO_4_, and incubated at room temperature for 10 min. The filter plates were then washed three times with 250 μl of 150 mM H_3_PO_4_ and once with 20 μl of 100% ethanol, dried for 30 min at 40 °C. After that, 50 μl of scintillator were added to each well and incorporation of ^33^Pi (“counting of cpm”) was determined with a microplate scintillation counter (Microbeta, Perkin Elmer).

For evaluation of the results of the glass-fiber filter assays, the autophosphorylation activity of each kinase had previously been determined in two independent experiments and mean autophosphorylation values (in cpm) had been calculated for each kinase at a given input of radioactivity (Table 1, column B). The background value of the protein sample was subtracted from each raw value (Table 1, ‘A–C’ column). The subtracted values were then normalized to the intrinsic autophosphorylation values for each kinase (Supplementary file 1, activity ratio column). A ratio value > 3 was considered significant phosphorylation of the substrate.

### *In vitro* kinase activity assay for TG-2

The kinase activity of recombinant TG-2 on paxillin was performed through a commercial service by ProQinase. The results were monitored by autoradiography of protein samples analyzed by SDS-PAGE. The reaction mixture containing the kinase and the substrate was pipetted into 1.5 ml eppendorf tubes in the following order: 10 μl 2.5x Standard Assay Buffer, 7.5 μl Substrate in 50 mM HEPES pH 7.5, 5 μl recombinant kinase in 1x Kinase Dilution Buffer, and 2.5 μl ATP + tracer (32P-γ-ATP in H2O approx 4.5*106 cpm/sample). The total volume of the reaction mixture was 25 μl, and the final assay concentration of ATP was 10 μM.

The samples were mixed and incubated at 30 °C for 60 min. The reaction was stopped by addition of 10 μl of 4x SDS sample-buffer and incubation at 95 °C for 5 min. Twenty microliters of the samples were loaded on a 4–12% Bis-Tris PAGE gel and analyzed by electrophoresis. The gel was stained with colloidal coomassie blue solution, dried and visualized by exposed to X-ray film (Kodak BioMax MR) for various periods of time.

### Cell free *in vitro* immunoprocipitation

Recombinant human proteins TG-2 (R&D system), JNK (Santa Cruz) and paxillin (Biolegend) were pre-cleared in 1.5 ml tubes by streptavidin agarose resin for 1 hr at 4 °C. The tubes were centrifuged at 1,000 g for 1 min at 4 °C and the supernatant was transferred to new tubes, and the streptavidin agarose beads were discarded. JNK (5 µg) and paxillin (1 µg) were mixed with 1 µg, 2.5 µg or 5 µg of TG-2 respectively in 1.5 ml tubes. Biotinylated anti-paxillin (10 µg) was added to each tube, and PBS was added to make each tube to 200 µl final volume. The tubes were incubated at 4 °C for overnight with end-over-end mixing. The following day, the same amount of streptavidin agarose resin used for pre-clearing was added to each tube and the tubes were incubated at 4 °C for 2 hrs. The supernatant was removed from each tube and resin beads were washed with 500 µl of cold PBS for 5 times. Fifty microliter of 2x protein loading dye was added to each tube and the tubes were heated at 95 °C for 10 min. The same volume from different samples was used for immuno blot with anti-TG-2, anti-JNK and anti-paxillin antibodies (Table 2).

### Data analysis

Unless specified otherwise above, at least 3 independent replicates were used for each type of experiment to ensure reproducibility. Statistical significance was determined at the level of alpha = 0.05. The normality of the data was first tested using Shapiro–Wilk test. For data comparing between two groups U test was used. To compare statistical differences for several groups ANOVA was done, followed by post-hoc comparisons, with p ≤ 0.05 being considered significant.

## Electronic supplementary material

Supplementary data
