# Supplementary material for: Mechanistic role of transglutaminase-2 in focal adhesions
Source: Sci Rep. 2018 Aug 17;8:12370. doi: 10.1038/s41598-018-30172-8 (PMC6098073; doi:10.1038/s41598-018-30172-8)
Supplement: Supplementary file 1 — Supplementary data [file 41598_2018_30172_MOESM1_ESM.docx]

**Mechanistic role of transglutaminase-2 in focal adhesions**

**Evelyn Png^a^, Hou Aihua^a,c^, Louis Tong^a,b,c,d*^**

^a^ Ocular Surface Research Group, Singapore Eye Research Institute, Singapore 168751,

^b^ Department of Cornea and External Eye Disease, Singapore National Eye Center, 11 Third Hospital Avenue, Singapore 168751.

^c^ Duke-NUS Graduate Medical School, Singapore,

^d^ Yong Loo Lin School of Medicine, National University of Singapore, Singapore

Running title: TG-2 binds non-covalently to Paxillin

* Address correspondence to: Dr Louis Tong, 20 College Road, The Academia, Level 6, Discovery Tower, Singapore 169856. Telephone: +65-6576 7213. Fax: +65-6225 2568. E-mail: [Louis.tong.h.t@snec.com.sg](mailto:Louis.tong.h.t@snec.com.sg)

**Keywords:** Transglutaminase, focal adhesion, cornea, cell adhesion, JNK

The authors declare no conflict of interest.

Supplementary File 1

Phosphorylation profile of protein “Human Paxillin” in 190 Ser/Thr kinase assays; singlicate measurement (Sample protein concentration: 5µg/50µl, all values in cpm)

|  |  | **A** | | **B** | |  | | **C** | |  | |  | |  |
| --- | --- | --- | --- | --- | --- | --- | --- | --- | --- | --- | --- | --- | --- | --- |
| **Kinase** | **Enzyme, ng/well** | **Activity raw values** | | **Kinase autophos., normalized mean n=3** | | Kinase autophos., SD | | Substrate-BG, mean of 2 singlicates | | **Activity values, corrected (A-C)** | | **Activity Ratio (A-C)/B** | | **Normalised to 10ng enzyme** |
| **Serine/ Threonine Kinases** |  |  |  | |  | |  | |  | |  | |  | |
| **CAMK2D** | 1 | 5262 | | **298** | | 63 | | 394 | | **4868** | | **16.36** | | 163.5752691 |
| **PRKG2** | 1 | 2613 | | **151** | | 42 | | 394 | | **2219** | | **14.73** | | 147.3392139 |
| **DYRK1B** | 2 | 6317 | | **81** | | 63 | | 394 | | **5923** | | **73.12** | | 365.609895 |
| **PIM1** | 2 | 2449 | | **137** | | 17 | | 394 | | **2055** | | **15.00** | | 75.00916455 |
| **p38-delta** | 2 | 2357 | | **138** | | 15 | | 394 | | **1963** | | **14.19** | | 70.92660777 |
| **CLK2** | 2 | 2281 | | **137** | | 41 | | 394 | | **1887** | | **13.73** | | 68.62734405 |
| **CAMK2A** | 2 | 1593 | | **130** | | 14 | | 394 | | **1199** | | **9.21** | | 46.04299917 |
| **NEK9** | 2 | 4798 | | **593** | | 22 | | 394 | | **4404** | | **7.43** | | 37.15912149 |
| **ASK1** | 2 | 2767 | | **374** | | 51 | | 394 | | **2373** | | **6.35** | | 31.74727145 |
| **RPS6KA6** | 2 | 946 | | **109** | | 31 | | 394 | | **552** | | **5.07** | | 25.32879651 |
| **CK1-epsilon** | 2.5 | 9899 | | **250** | | 59 | | 394 | | **9505** | | **38.03** | | 152.1223163 |
| **SNF1LK2** | 2.5 | 5041 | | **289** | | 11 | | 394 | | **4647** | | **16.09** | | 64.36241537 |
| **PKC-beta1** | 2.5 | 1841 | | **105** | | 31 | | 394 | | **1447** | | **13.80** | | 55.18802201 |
| **ROCK2** | 2.5 | 1490 | | **122** | | 7 | | 394 | | **1096** | | **8.97** | | 35.89474212 |
| **MYLK2** | 2.5 | 985 | | **92** | | 9 | | 394 | | **591** | | **6.41** | | 25.62877847 |
| **PKC-alpha** | 2.5 | 1612 | | **196** | | 98 | | 394 | | **1218** | | **6.21** | | 24.82155711 |
| **PKC-theta** | 2.5 | 737 | | **171** | | 79 | | 394 | | **343** | | **2.01** | | 8.034539804 |
| **DYRK3** | 3 | 8278 | | **316** | | 13 | | 394 | | **7884** | | **24.97** | | 83.2283247 |
| **MAP4K5** | 3 | 2265 | | **279** | | 46 | | 394 | | **1871** | | **6.70** | | 22.33923375 |
| **BRSK1** | 3 | 416 | | **139** | | 54 | | 394 | | **22** | | **0.16** | | 0.528516733 |
| **p38-beta** | 3 | 408 | | **112** | | 82 | | 394 | | **14** | | **0.12** | | 0.416303808 |
| **PIM3** | 4 | 10196 | | **329** | | 14 | | 394 | | **9802** | | **29.79** | | 74.47257389 |
| **ROCK1** | 4 | 2511 | | **131** | | 56 | | 394 | | **2117** | | **16.12** | | 40.3054618 |
| **ZAK** | 4 | 3011 | | **418** | | 70 | | 394 | | **2617** | | **6.27** | | 15.66351714 |
| **MAP4K2** | 4 | 896 | | **280** | | 23 | | 394 | | **502** | | **1.80** | | 4.488733211 |
| **NEK4** | 4 | 902 | | **415** | | 67 | | 394 | | **508** | | **1.22** | | 3.060237127 |
| **RON** | 4 | 542 | | **122** | | 47 | | 394 | | **148** | | **1.22** | | 3.043446911 |
| **JNK1** | 5 | 3986 | | **43** | | 33 | | 394 | | **3592** | | **84.34** | | 168.6778177 |
| **PKA** | 5 | 7600 | | **87** | | 14 | | 394 | | **7206** | | **82.43** | | 164.8663736 |
| **MARK2** | 5 | 24606 | | **330** | | 34 | | 394 | | **24212** | | **73.30** | | 146.6062914 |
| **TSSK1** | 5 | 14094 | | **204** | | 134 | | 394 | | **13700** | | **67.25** | | 134.4956351 |
| **CK1-delta** | 5 | 12435 | | **281** | | 24 | | 394 | | **12041** | | **42.86** | | 85.71246829 |
| **TBK1** | 5 | 18074 | | **431** | | 37 | | 394 | | **17680** | | **41.03** | | 82.06631972 |
| **CK1-gamma1** | 5 | 8253 | | **255** | | 7 | | 394 | | **7859** | | **30.81** | | 61.62292669 |
| **RPS6KA3** | 5 | 2888 | | **86** | | 19 | | 394 | | **2494** | | **29.15** | | 58.29160674 |
| **MAP4K4** | 5 | 3837 | | **144** | | 50 | | 394 | | **3443** | | **23.83** | | 47.65713611 |
| **PKC-beta2** | 5 | 2833 | | **108** | | 13 | | 394 | | **2439** | | **22.53** | | 45.06715624 |
| **GRK4** | 5 | 5083 | | **223** | | 135 | | 394 | | **4689** | | **20.99** | | 41.97315687 |
| **JNK3** | 5 | 1862 | | **100** | | 17 | | 394 | | **1468** | | **14.70** | | 29.39484328 |
| **p38-gamma** | 5 | 1786 | | **108** | | 25 | | 394 | | **1392** | | **12.95** | | 25.89216559 |
| **GRK5** | 5 | 1450 | | **87** | | 3 | | 394 | | **1056** | | **12.20** | | 24.40102896 |
| **CK1-gamma3** | 5 | 2946 | | **222** | | 91 | | 394 | | **2552** | | **11.48** | | 22.96681459 |
| **LRRK G2019S** | 5 | 1965 | | **221** | | 7 | | 394 | | **1571** | | **7.12** | | 14.24434215 |
| **GRK7** | 5 | 1033 | | **95** | | 53 | | 394 | | **639** | | **6.75** | | 13.50983883 |
| **PHKG1** | 5 | 1020 | | **96** | | 0 | | 394 | | **626** | | **6.49** | | 12.98763251 |
| **NEK1** | 5 | 4484 | | **936** | | 311 | | 394 | | **4090** | | **4.37** | | 8.736599688 |
| **MST1** | 5 | 2506 | | **522** | | 62 | | 394 | | **2112** | | **4.05** | | 8.096123663 |
| **JNK2** | 5 | 714 | | **132** | | 64 | | 394 | | **320** | | **2.43** | | 4.857427093 |
| **PRKD2** | 5 | 683 | | **440** | | 14 | | 394 | | **289** | | **0.66** | | 1.313232392 |
| **PRKX** | 10 | 5872 | | **77** | | 51 | | 394 | | **5478** | | **71.14** | | 71.14109972 |
| **MAPKAPK3** | 10 | 1632 | | **18** | | 2 | | 394 | | **1238** | | **67.28** | | 67.27991525 |
| **GRK3** | 10 | 6065 | | **112** | | 2 | | 394 | | **5671** | | **50.75** | | 50.75476634 |
| **CK1-gamma2** | 10 | 14823 | | **300** | | 18 | | 394 | | **14429** | | **48.15** | | 48.15168637 |
| **MINK1** | 10 | 6843 | | **183** | | 90 | | 394 | | **6449** | | **35.22** | | 35.22115499 |
| **DYRK1A** | 10 | 14224 | | **628** | | 108 | | 394 | | **13830** | | **22.03** | | 22.02783978 |
| **RPS6KA1** | 10 | 4549 | | **189** | | 14 | | 394 | | **4155** | | **21.98** | | 21.97849972 |
| **CDK4/CycD3** | 10 | 1339 | | **45** | | 21 | | 394 | | **945** | | **21.07** | | 21.06640405 |
| **ERK2** | 10 | 1855 | | **85** | | 25 | | 394 | | **1461** | | **17.15** | | 17.14547611 |
| **CDK2/CycE** | 10 | 2598 | | **156** | | 30 | | 394 | | **2204** | | **14.17** | | 14.1696478 |
| **NLK** | 10 | 2367 | | **154** | | 88 | | 394 | | **1973** | | **12.85** | | 12.84973658 |
| **ACV-R1B** | 10 | 2432 | | **173** | | 26 | | 394 | | **2038** | | **11.78** | | 11.77824848 |
| **CDK3/CycE** | 10 | 2464 | | **199** | | 15 | | 394 | | **2070** | | **10.39** | | 10.38786261 |
| **PKC-epsilon** | 10 | 2438 | | **208** | | 53 | | 394 | | **2044** | | **9.82** | | 9.817638839 |
| **RPS6KA2** | 10 | 3471 | | **372** | | 37 | | 394 | | **3077** | | **8.28** | | 8.281286805 |
| **CLK3** | 10 | 2144 | | **213** | | 29 | | 394 | | **1750** | | **8.21** | | 8.205328631 |
| **PHKG2** | 10 | 4629 | | **550** | | 222 | | 394 | | **4235** | | **7.70** | | 7.695669792 |
| **TGFB-R1** | 10 | 1924 | | **206** | | 54 | | 394 | | **1530** | | **7.42** | | 7.418483141 |
| **PKC-eta** | 10 | 5120 | | **649** | | 20 | | 394 | | **4726** | | **7.28** | | 7.276710793 |
| **MST2** | 10 | 4854 | | **708** | | 139 | | 394 | | **4460** | | **6.30** | | 6.299204572 |
| **CAMKK2** | 10 | 4308 | | **640** | | 63 | | 394 | | **3914** | | **6.11** | | 6.112935504 |
| **WNK3** | 10 | 1902 | | **266** | | 1 | | 394 | | **1508** | | **5.66** | | 5.664019775 |
| **RAF1 DYDY** | 10 | 1457 | | **191** | | 8 | | 394 | | **1063** | | **5.56** | | 5.559579091 |
| **CHK2** | 10 | 2291 | | **415** | | 119 | | 394 | | **1897** | | **4.57** | | 4.569181646 |
| **DAPK2 +CaM)** | 10 | 1462 | | **257** | | 5 | | 394 | | **1068** | | **4.16** | | 4.156725166 |
| **PKC-gamma** | 10 | 1178 | | **213** | | 6 | | 394 | | **784** | | **3.67** | | 3.673456602 |
| **AKT3** | 10 | 1662 | | **363** | | 29 | | 394 | | **1268** | | **3.49** | | 3.493673732 |
| **EIF2AK2** | 10 | 1873 | | **451** | | 42 | | 394 | | **1479** | | **3.28** | | 3.280331631 |
| **IRAK4** | 10 | 1218 | | **274** | | 25 | | 394 | | **824** | | **3.01** | | 3.008438616 |
| **DAPK3** | 10 | 1412 | | **398** | | 3 | | 394 | | **1018** | | **2.56** | | 2.557960087 |
| **CDC42BPA** | 10 | 573 | | **87** | | 4 | | 394 | | **179** | | **2.06** | | 2.057448197 |
| **NEK3** | 10 | 1844 | | **863** | | 146 | | 394 | | **1450** | | **1.68** | | 1.679400845 |
| **TGFB-R2** | 10 | 704 | | **309** | | 71 | | 394 | | **310** | | **1.00** | | 1.002972887 |
| **p38-alpha** | 10 | 439 | | **92** | | 29 | | 394 | | **45** | | **0.49** | | 0.491328546 |
| **PRKG1** | 12.5 | 1562 | | **68** | | 18 | | 394 | | **1168** | | **17.08** | | 13.66677166 |
| **CDK5/p25NCK** | 15 | 13798 | | **265** | | 66 | | 394 | | **13404** | | **50.62** | | 33.74890374 |
| **GRK6** | 15 | 7032 | | **225** | | 50 | | 394 | | **6638** | | **29.53** | | 19.68994122 |
| **CDK9/CycT** | 15 | 12255 | | **633** | | 141 | | 394 | | **11861** | | **18.73** | | 12.48998253 |
| **CDK5/p35NCK** | 15 | 8627 | | **514** | | 48 | | 394 | | **8233** | | **16.01** | | 10.67342508 |
| **CDK1/CycA** | 15 | 3510 | | **240** | | 2 | | 394 | | **3116** | | **12.97** | | 8.644444829 |
| **PAK7** | 15 | 655 | | **224** | | 43 | | 394 | | **261** | | **1.16** | | 0.775733906 |
| **PAK1** | 15 | 355 | | **170** | | 51 | | 394 | | **-39** | | **-0.23** | | -0.152640821 |
| **IKK-epsilon** | 20 | 26037 | | **619** | | 42 | | 394 | | **25643** | | **41.41** | | 20.70578823 |
| **HIPK1** | 20 | 4952 | | **147** | | 23 | | 394 | | **4558** | | **30.96** | | 15.48105019 |
| **HIPK3** | 20 | 3172 | | **119** | | 57 | | 394 | | **2778** | | **23.41** | | 11.7051582 |
| **NEK6** | 20 | 5830 | | **283** | | 111 | | 394 | | **5436** | | **19.22** | | 9.611154599 |
| **ERK1** | 20 | 2373 | | **103** | | 35 | | 394 | | **1979** | | **19.19** | | 9.597324311 |
| **mTOR** | 20 | 6578 | | **452** | | 74 | | 394 | | **6184** | | **13.67** | | 6.833945087 |
| **SGK2** | 20 | 4414 | | **401** | | 34 | | 394 | | **4020** | | **10.03** | | 5.016842769 |
| **TAOK2** | 20 | 6160 | | **654** | | 100 | | 394 | | **5766** | | **8.81** | | 4.406756638 |
| **DCAMKL2** | 20 | 3620 | | **558** | | 142 | | 394 | | **3226** | | **5.78** | | 2.890304426 |
| **ACV-RL1** | 20 | 4374 | | **894** | | 126 | | 394 | | **3980** | | **4.45** | | 2.224850961 |
| **EIF2AK3** | 20 | 6545 | | **1606** | | 64 | | 394 | | **6151** | | **3.83** | | 1.914495145 |
| **IRAK1** | 20 | 8952 | | **2468** | | 432 | | 394 | | **8558** | | **3.47** | | 1.733470179 |
| **CK2-alpha1** | 20 | 1845 | | **519** | | 255 | | 394 | | **1451** | | **2.80** | | 1.398043977 |
| **PDK1** | 20 | 1247 | | **332** | | 15 | | 394 | | **853** | | **2.57** | | 1.285359539 |
| **ACV-R1** | 20 | 1778 | | **612** | | 175 | | 394 | | **1384** | | **2.26** | | 1.130028247 |
| **SRPK2** | 20 | 1236 | | **389** | | 181 | | 394 | | **842** | | **2.16** | | 1.082075514 |
| **MST3** | 20 | 2692 | | **1200** | | 255 | | 394 | | **2298** | | **1.92** | | 0.95781898 |
| **PAK3** | 20 | 1132 | | **462** | | 75 | | 394 | | **738** | | **1.60** | | 0.799310872 |
| **PKC-mu** | 20 | 1290 | | **585** | | 9 | | 394 | | **896** | | **1.53** | | 0.765415442 |
| **PRK2** | 20 | 721 | | **247** | | 1 | | 394 | | **327** | | **1.32** | | 0.66170929 |
| **MAPKAPK5** | 20 | 483 | | **94** | | 60 | | 394 | | **89** | | **0.95** | | 0.473289916 |
| **NEK7** | 25 | 22181 | | **304** | | 86 | | 394 | | **21787** | | **71.64** | | 28.65790576 |
| **TSF1** | 25 | 11932 | | **282** | | 93 | | 394 | | **11538** | | **40.89** | | 16.35497184 |
| **AKT1** | 25 | 3419 | | **216** | | 35 | | 394 | | **3025** | | **13.99** | | 5.595784009 |
| **RPS6KA5** | 25 | 3050 | | **215** | | 33 | | 394 | | **2656** | | **12.38** | | 4.951375191 |
| **CDK1CycB1** | 25 | 6440 | | **503** | | 98 | | 394 | | **6046** | | **12.02** | | 4.806564947 |
| **B-RAF VE** | 25 | 2088 | | **175** | | 30 | | 394 | | **1694** | | **9.66** | | 3.864410389 |
| **B-RAF wt** | 25 | 3090 | | **312** | | 20 | | 394 | | **2696** | | **8.65** | | 3.459232319 |
| **CDK4/CycD1** | 25 | 1091 | | **124** | | 89 | | 394 | | **697** | | **5.61** | | 2.245368138 |
| **NEK11** | 25 | 3741 | | **827** | | 100 | | 394 | | **3347** | | **4.05** | | 1.618104512 |
| **TSK2** | 25 | 6792 | | **1688** | | 364 | | 394 | | **6398** | | **3.79** | | 1.515919181 |
| **PRK1** | 25 | 2275 | | **575** | | 42 | | 394 | | **1881** | | **3.27** | | 1.309051232 |
| **PKC-delta** | 25 | 4345 | | **1304** | | 18 | | 394 | | **3951** | | **3.03** | | 1.212297026 |
| **CDC42BPB** | 25 | 1235 | | **414** | | 60 | | 394 | | **841** | | **2.03** | | 0.813481922 |
| **STK17A** | 25 | 1534 | | **630** | | 31 | | 394 | | **1140** | | **1.81** | | 0.724005985 |
| **PAK6** | 25 | 1302 | | **625** | | 167 | | 394 | | **908** | | **1.45** | | 0.581519211 |
| **SRPK1** | 25 | 849 | | **347** | | 80 | | 394 | | **455** | | **1.31** | | 0.524755225 |
| **STK23** | 25 | 2990 | | **4654** | | 737 | | 394 | | **2596** | | **0.56** | | 0.223112234 |
| **PAK2** | 25 | 426 | | **171** | | 17 | | 394 | | **32** | | **0.19** | | 0.074896204 |
| **PLK3** | 30 | 8825 | | **611** | | 127 | | 394 | | **8431** | | **13.79** | | 4.596052506 |
| **WNK2** | 40 | 1969 | | **279** | | 78 | | 394 | | **1575** | | **5.65** | | 1.412805672 |
| **DAPK1** | 40 | 1501 | | **792** | | 87 | | 394 | | **1107** | | **1.40** | | 0.349390263 |
| **GRK2** | 50 | 16225 | | **232** | | 83 | | 394 | | **15831** | | **68.17** | | 13.63375058 |
| **CDK2/CycA** | 50 | 27128 | | **621** | | 40 | | 394 | | **26734** | | **43.03** | | 8.606452242 |
| **CDK7CycH** | 50 | 11528 | | **296** | | 97 | | 394 | | **11134** | | **37.60** | | 7.519819072 |
| **CDK8/CycC** | 50 | 2779 | | **69** | | 26 | | 394 | | **2385** | | **34.80** | | 6.959159135 |
| **GSK3-beta** | 50 | 14325 | | **426** | | 144 | | 394 | | **13931** | | **32.68** | | 6.535139213 |
| **PLK1** | 50 | 33010 | | **1750** | | 23 | | 394 | | **32616** | | **18.63** | | 3.726891276 |
| **LIMK1** | 50 | 21158 | | **1130** | | 7 | | 394 | | **20764** | | **18.38** | | 3.676094124 |
| **GSK3-alpha** | 50 | 56578 | | **3524** | | 84 | | 394 | | **56184** | | **15.94** | | 3.188866597 |
| **CDK1/CycE** | 50 | 3784 | | **248** | | 41 | | 394 | | **3390** | | **13.69** | | 2.738869159 |
| **PASK** | 50 | 26320 | | **2785** | | 77 | | 394 | | **25926** | | **9.31** | | 1.861945038 |
| **CK2-alpha2** | 50 | 1435 | | **139** | | 60 | | 394 | | **1041** | | **7.48** | | 1.496647689 |
| **RIPK2** | 50 | 3639 | | **435** | | 22 | | 394 | | **3245** | | **7.47** | | 1.49353865 |
| **IKK-alpha** | 50 | 8111 | | **1406** | | 129 | | 394 | | **7717** | | **5.49** | | 1.097433617 |
| **MEK1 wt** | 50 | 1367 | | **200** | | 7 | | 394 | | **973** | | **4.87** | | 0.974185647 |
| **RPS6KA4** | 50 | 3979 | | **874** | | 92 | | 394 | | **3585** | | **4.10** | | 0.820297331 |
| **TAOK3** | 50 | 3788 | | **848** | | 182 | | 394 | | **3394** | | **4.00** | | 0.800285427 |
| **MKK6SDTD** | 50 | 941 | | **145** | | 2 | | 394 | | **547** | | **3.77** | | 0.754840218 |
| **PIM2** | 50 | 1299 | | **255** | | 79 | | 394 | | **905** | | **3.54** | | 0.708551214 |
| **CHK1** | 50 | 20145 | | **6033** | | 214 | | 394 | | **19751** | | **3.27** | | 0.65477374 |
| **STK33** | 50 | 4168 | | **1182** | | 75 | | 394 | | **3774** | | **3.19** | | 0.638431425 |
| **SGK3** | 50 | 2264 | | **767** | | 196 | | 394 | | **1870** | | **2.44** | | 0.487371911 |
| **PKC-nu** | 50 | 3016 | | **1269** | | 141 | | 394 | | **2622** | | **2.07** | | 0.413347277 |
| **NEK2** | 50 | 10193 | | **5041** | | 660 | | 394 | | **9799** | | **1.94** | | 0.388774284 |
| **SNK** | 50 | 4984 | | **2529** | | 344 | | 394 | | **4590** | | **1.81** | | 0.36292205 |
| **PKC-zeta** | 50 | 5228 | | **2692** | | 37 | | 394 | | **4834** | | **1.80** | | 0.35913113 |
| **PKC-iota** | 50 | 921 | | **312** | | 57 | | 394 | | **527** | | **1.69** | | 0.337390936 |
| **VRK1** | 50 | 3245 | | **2384** | | 545 | | 394 | | **2851** | | **1.20** | | 0.239209806 |
| **PAK4** | 50 | 1222 | | **792** | | 75 | | 394 | | **828** | | **1.05** | | 0.209131836 |
| **SGK1** | 50 | 555 | | **177** | | 65 | | 394 | | **161** | | **0.91** | | 0.181418173 |
| **Aurora-A** | 50 | 2807 | | **2708** | | 645 | | 394 | | **2413** | | **0.89** | | 0.178221048 |
| **S6K** | 50 | 510 | | **219** | | 11 | | 394 | | **116** | | **0.53** | | 0.106031632 |
| **CAMK4** | 75 | 6672 | | **2082** | | 54 | | 394 | | **6278** | | **3.02** | | 0.402065662 |
| **HRI** | 80 | 7145 | | **1904** | | 297 | | 394 | | **6751** | | **3.55** | | 0.443157307 |
| **DMPK** | 80 | 374 | | **302** | | 89 | | 394 | | **-20** | | **-0.07** | | -0.0082858 |
| **CAMK2B** | 100 | 5685 | | **230** | | 66 | | 394 | | **5291** | | **22.98** | | 2.297534166 |
| **MARK3** | 100 | 72504 | | **4680** | | 557 | | 394 | | **72110** | | **15.41** | | 1.54093949 |
| **MARK1** | 100 | 42294 | | **2847** | | 682 | | 394 | | **41900** | | **14.72** | | 1.471656895 |
| **CAMK1D** | 100 | 11069 | | **2275** | | 401 | | 394 | | **10675** | | **4.69** | | 0.469271203 |
| **CK1-alpha1** | 100 | 12277 | | **3182** | | 1014 | | 394 | | **11883** | | **3.73** | | 0.373487214 |
| **S6K-beta** | 100 | 2558 | | **699** | | 150 | | 394 | | **2164** | | **3.10** | | 0.30955203 |
| **TTK** | 100 | 11573 | | **4827** | | 300 | | 394 | | **11179** | | **2.32** | | 0.231592583 |
| **SAK** | 100 | 9396 | | **4227** | | 134 | | 394 | | **9002** | | **2.13** | | 0.212946566 |
| **MELK** | 100 | 592 | | **103** | | 44 | | 394 | | **198** | | **1.92** | | 0.191912638 |
| **MST4** | 100 | 4496 | | **2219** | | 463 | | 394 | | **4102** | | **1.85** | | 0.184829733 |
| **IKK-beta** | 100 | 9378 | | **5428** | | 1246 | | 394 | | **8984** | | **1.66** | | 0.165505513 |
| **Aurora-C** | 100 | 1908 | | **998** | | 324 | | 394 | | **1514** | | **1.52** | | 0.151705744 |
| **ARK5** | 100 | 10963 | | **9809** | | 2605 | | 394 | | **10569** | | **1.08** | | 0.10774848 |
| **Aurora-B** | 100 | 2155 | | **1808** | | 232 | | 394 | | **1761** | | **0.97** | | 0.097380818 |
| **CDK6/CycD1** | 200 | 1013 | | **120** | | 1 | | 394 | | **619** | | **5.17** | | 0.258686032 |
| **PBK** | 200 | 3339 | | **703** | | 13 | | 394 | | **2945** | | **4.19** | | 0.209403119 |
| **AKT2** | 200 | 13412 | | **3893** | | 738 | | 394 | | **13018** | | **3.34** | | 0.16720184 |
| **AMPK-alpha1** | 200 | 6155 | | **2580** | | 576 | | 394 | | **5761** | | **2.23** | | 0.111653888 |
| **SNARK** | 200 | 9517 | | **8641** | | 790 | | 394 | | **9123** | | **1.06** | | 0.052788258 |
| **COT** | 300 | 6201 | | **3368** | | 32 | | 394 | | **5807** | | **1.72** | | 0.057466094 |
| **NIK** | 350 | 8704 | | **5919** | | 767 | | 394 | | **8310** | | **1.40** | | 0.040112914 |
| **PCTAIRE1** | 400 | 3343 | | **2260** | | 481 | | 394 | | **2949** | | **1.30** | | 0.032617265 |
| **CLK1** | 400 | 7446 | | **6511** | | 1768 | | 394 | | **7052** | | **1.08** | | 0.027077389 |
